# Immature CD10^low^ blood neutrophils are enriched in people with multiple sclerosis

**DOI:** 10.3389/fimmu.2026.1782621

**Published:** 2026-04-29

**Authors:** Luke W. Garratt, Alice A. White, Craig Schofield, Jonatan Leffler, Prue H. Hart, Marzena J. Fabis-Pedrini, Allan G. Kermode, Anne Brüstle, Stephanie Trend

**Affiliations:** 1 Wal-Yan Respiratory Research Centre, The Kids Research Institute Australia, Perth, WA, Australia; 2 Centre for Child Health Research, University of Western Australia, Perth, WA, Australia; 3 Translational Immunology Team, The Kids Research Institute Australia, Perth, WA, Australia; 4 Demyelinating Diseases Research Group, Perron Institute for Neurological and Translational Science, University of Western Australia, Perth, WA, Australia; 5 Personalised Medicine Centre, Health Futures Institute, Murdoch University, Perth, WA, Australia; 6 School of Biomedical Sciences, University of Western Australia, Perth, WA, Australia; 7 Institute for Immunology and Infectious Disease, Murdoch University, Perth, WA, Australia; 8 John Curtin School of Medical Research, Australian National University, Canberra, ACT, Australia

**Keywords:** CD10, flow cytometry, multiple sclerosis, neutrophil, pathogenesis

## Abstract

**Introduction:**

Neutrophils are proposed to contribute to inflammation at the onset of multiple sclerosis (MS). However, as neutrophils must be analysed rapidly following blood collection, their characterisation remains challenging, and the biology of neutrophils during episodes of MS is poorly understood. Neutrophils can comprise of several subpopulations and diverse phenotypes that are modified across health states and tissues. We hypothesised that neutrophil subpopulations would significantly differ in abundance between people with MS and controls.

**Method:**

Our pilot study applied flow cytometry to analyse phenotypes of neutrophils present in the peripheral blood of 10 people with recently active MS or clinically isolated syndrome (CIS) and 12 control participants.

**Results:**

Using both unsupervised and supervised analyses of flow cytometry data, we identified that CD10^low^ neutrophils were significantly enriched in the blood of people with CIS and MS compared with controls. These CD10^low^ neutrophils featured decreased CD16 and CD11b expression, with CD184 expression absent, suggesting they were an immature neutrophil population. In people with MS, the proportions of CD10^low^ neutrophils were non-significantly correlated with expanded disability status scores (p=0.06).

**Discussion:**

These findings point to immature CD10^low^ blood neutrophils as a population of interest to active MS disease, whose functions should be studied in greater detail in context of MS pathology and biomarkers.

## 1 Introduction

Multiple sclerosis (MS) is an immune-mediated condition that involves episodes of demyelination in the central nervous system (CNS). In addition to Epstein-Barr Virus (EBV) infection (1), a number of other risk factors for MS have been identified, including >200 genetic polymorphisms predominantly associated with immune regulation (2). Although MS is primarily thought to be driven by T and B cells (3), neutrophils are emerging as a potential mediator of early disease (4). There is evidence that neutrophils may precede the influx of B cells into the CNS during initiation of MS (5), but their numbers in the CNS decline as the disease progresses (6).

Hypothesised roles for neutrophils in MS pathogenesis include presentation of antigens to adaptive immune cells, production of inflammatory chemokines, contributing to the breakdown of the blood-brain barrier, and production of neutrophil extracellular traps (NETs) (7). Furthermore, an increased neutrophil to lymphocyte ratio (NLR) in peripheral blood is a proposed biomarker of active MS episodes (8, 9). Compared with controls, peripheral blood neutrophils of people with MS typically exhibit higher expression of activation markers such as TLR2, CD43, fMLP receptor, and IL-8 receptor (10, 11). In addition, proteins derived from neutrophil activation, such as neutrophil elastase, are regularly observed to be higher in MS sera (11). Isolated neutrophils from people with MS also display increased responsiveness to activation compared to healthy individuals (10, 11). However, much remains unknown about whether specific neutrophil subpopulations are involved in MS.

It is mostly accepted that several neutrophil subpopulations exist, and that these may be associated with disease states (12). Despite this, phenotyping of neutrophils is generally inconsistent. Recently, we reported the first Optimized Multicolor Immunofluorescence Panel (OMIP) for phenotyping neutrophil populations, OMIP-100 (13). To gain further insight into the role of neutrophils in MS disease onset, we applied a near-final iteration of OMIP-100 to test the hypothesis that the blood of people with newly-diagnosed MS or CIS contained distinct or increased proportions of neutrophil subsets compared with controls. We also investigated whether unsupervised clustering analysis could identify unique neutrophil populations not currently captured by manual gating methods.

## 2 Methods

### 2.1 Study participant recruitment and sample collection

People presenting to the recruiting neurologist with radiologically isolated syndrome (RIS), clinically isolated syndrome CIS, or a new diagnosis of MS (2017 McDonald criteria), aged ≥18 years, were recruited through a private neurological clinic in Perth, Western Australia between November 2021 and March 2023. Control participants were recruited from the matching general population that met the following criteria: no history of autoimmunity, neurological disease or current infectious illness. Written informed consent was obtained prior to the collection of peripheral venous blood using K_2_EDTA Vacutainers (BD) and transported to the processing laboratory at The Kids Research Institute Australia. This study was approved by the University of Western Australia Human Research Ethics Committee (approval RA/4/20/5859) and the CAHS Human Research Ethics Committee (approval RGS1470). This study was carried out in accordance with the recommendations of the National Health and Medical Research Council of Australia’s National Statement on Ethical Conduct in Human Research 2007 (updated 2018).

A total of 22 participants were included (**Table 1**); 10 participants with acute demyelinating syndromes (RIS n=1, CIS n=2, MS n=7), and 12 controls. Given that the individual with RIS developed definite MS within 6 months of the sample collection, the cohort is referred to as ‘CIS/MS’ hereafter. Participants with CIS/MS were within 16 days (median) of a diagnostic MRI showing demyelinating lesions. For the 7 participants (6 MS, 1 CIS) with available clinical scores within 1 month prior to blood sample collection (maximum 18 days prior, median 1 day prior to blood collection), they had a median Expanded Disability Status Scale (EDSS) score of 1.5 (range 0–5). No participants had been treated with MS disease modifying therapies before the time of blood collection. One participant with CIS was being treated with corticosteroids, and one participant with MS had been treated with corticosteroids that ceased 1 month prior to sample collection. There were no significant differences between people with CIS/MS or controls in age, or proportions of females within each group (**Table 1**).

**Table 1:** Clinical phenotypes of donors participating in the study.

Variable	Control	CIS/MS	P-value
Age (median [range])	41 (26–51)	32 (18–56)	0.28
Sex (n [%])			1.0
Female	6 (50%)	5 (50%)	
Male	6 (50%)	5 (50%)	
EDSS (median [range]; n=7)	n/a	1.5 (0–5)	n/a
Diagnosis	n/a		n/a
*RIS*		1	
*CIS*		2	
*New MS*		7	
Days since MRI with newest* lesion observed (median [range])	n/a		n/a
All		16 (0–48)	
*RIS*		13 (13–13)	
*CIS*		7 (4–10)	
*New MS*		20 (0–48)	
Days since first recorded demyelinating event (median [range])	n/a		n/a
All		19.5 (4–344)	
*RIS*		13 (13–13)	
*CIS*		7 (4–10)	
*New MS*		21 (5–344)	

EDSS scores were only included for participants with a clinical assessment within 1 month of the blood draw (n=7). *In participants with MS with prior demyelinating events captured by MRI (n=3).

### 2.2 Flow cytometry staining and data acquisition

Whole blood samples received within three hours of venepuncture were prepared for analysis by flow cytometry, applying an iteration of a published panel for phenotyping neutrophil populations (13). Briefly, 20 µL of whole blood was mixed with phosphate buffered saline (PBS; 50 µL) and pre-treated with an Fc receptor blocking solution (Human TruStain FcX Fc Block; BioLegend; San Diego, CA) combined with an FVS575V viability dye (BD Biosciences; North Ryde, Australia) diluted in PBS containing 2.5 mM EDTA. The samples were incubated for 10 min on ice protected from light. Subsequently, cells were incubated for 20 min on ice with a multicolour flow cytometry antibody panel designed to identify neutrophil subpopulations (shown in **Supplementary Table 1**). Cells were washed and treated with Lyse/Fix buffer (BD Biosciences) at 37°C for 10 min according to the manufacturer’s instructions. Following washing, cells were permeabilised with ice-cold Perm II buffer (BD Biosciences) and incubated for 20 min at 4°C. After washing, cells were incubated with intracellular stain for OLFM-4 (**Supplementary Table 1**; Intracellular Panel) for 30 min at RT protected from light. Once washed and resuspended in PBS-EDTA, samples were immediately acquired using a LSR Fortessa (BD Biosciences). Prior to sample acquisition, the cytometer was calibrated to +/- <11% of target Median fluorescence intensity (MFI) values on each fluorophore using Ultra Rainbow calibration 6 peak beads (Spherotech; ProSciTech, Thuringowa, Australia). At least 4,000 neutrophil events (CD15^hi^/CD45^lo^/CD16^+^) were collected from each sample.

### 2.3 Flow cytometry data analysis

Flow cytometry data was analysed using FlowJo software (version 10.6.2; BD Biosciences), and compensation was applied using single stain bead controls (acquired at the same time as clinical samples). Conventional gating was performed for quality control to exclude doublets, Brilliant Violet™ dye aggregates, non-viable cells and CD45^-^ cells (**Supplementary Figure 1**). All CD45^+^ events were exported from FlowJo as compensated .fcs files for each sample for dimensionality reduction analyses.

### 2.4 Dimensionality reduction and visualisation of flow data

The exported .fcs files containing all CD45^+^ events were analysed using R version 4.3.1 (2023–06–16) on an x86_64-pc-linux-gnu (64-bit) platform running Ubuntu 22.04.5 LTS. The data were read in using flowcore (version 2.12.2), combined and transformed using a logicle function in CATALYST (version 1.24.0) (14). The generated flowset was down-sampled to 25,000 CD45^+^ cells per sample. Flow Self-Organising Map (FlowSOM)-based clustering was then performed using all panel markers, excluding FSC, SSC and viability stain. An estimated 20 meta-clusters were generated to facilitate the detection of rare cell populations. Following Uniform Manifold Approximation and Projection (UMAP)-based dimensionality reduction, the 20 meta-clusters were visualised and annotated based on the expression of phenotypic markers.

### 2.5 Manual gating of neutrophil populations

Neutrophils were identified amongst live CD45^+^ whole blood cell populations as CD15^high^/CD45^low^/CD16^+^ (**Supplementary Figure 1**). This was modified from the approach outlined in the OMIP-100 (13) due to different allocation of fluorophores to CD66b. Following the identification of CD15^high^/CD45^low^/CD16^+^ cells, downstream gating approaches were the same as OMIP-100. We also further added gates to quantify CD11b^-^ cells and CD11b^+^ cells. Eosinophils were excluded by their lack of CD16 expression. The MFI of all markers was obtained for the whole neutrophil population, as well as the proportions of gated subset populations. Gating used for distinguishing between positive and negative populations for markers on neutrophils, including CD10, were confirmed by using the mononuclear cell populations as a biological reference.

### 2.6 Statistical methods

The distributions of continuous variables were tested for normality using Shapiro-Wilk tests, which indicated data were not normally distributed. Therefore, comparisons between groups for continuous variables were made using two-sided Mann-Whitney tests for unpaired data, and using Wilcoxon matched-pairs signed rank test for paired data with the Holm-Šídák correction for multiple testing applied. Comparisons of males and females in the control and CIS/MS groups were made using Kruskal Wallis tests (with Dunn’s correction for multiple comparisons); correlation between continuous variables was conducted using Spearman correlation, using Prism software (version 10.4.0; GraphPad, San Diego, CA). Comparisons between groups of categorical variables (i.e. sex) were made using Fisher’s exact test in R. Linear regression modelling was performed using the lm function in R (15), with CD10^low^ neutrophil cluster proportions as the dependent variable, and EDSS, age, sex and an interaction of sex*EDSS as explanatory terms. P-values <0.05 were considered statistically significant in all tests.

## 3 Results

### 3.1 Neutrophil phenotypes in whole blood

**Figure 1:**
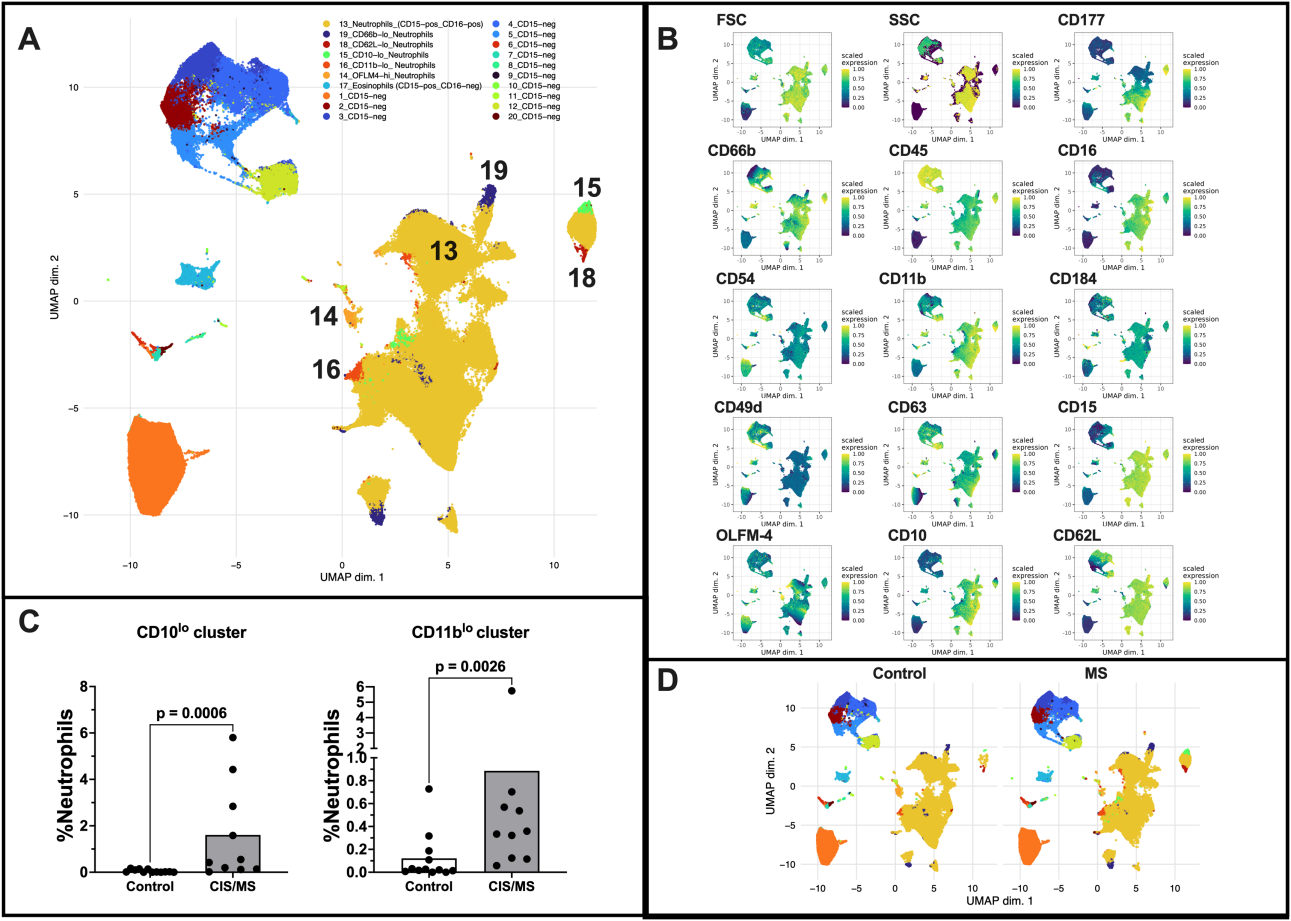
**(A)** Visualisation of cell metaclusters generated using CD45^+^ cell data including both the CIS/MS and control cohorts, through uniform manifold approximation and projection (UMAP) dimensionality reduction. Colours indicate distinct metaclusters (1–20) as shown in the figure legend. Neutrophil metaclusters are additionally labelled on the figure with the metacluster number for emphasis. **(B)** UMAP visualisation of the scaled expression of individual cellular markers in cell metaclusters identified. **(C)** CD10^lo^ and CD11b^lo^ neutrophil cluster proportions (% of total neutrophils) in controls and people with CIS/MS. P-values were obtained from Mann-Whitney tests, and are shown on the figures, with median values indicated by the shaded bar and individual values shown as circles. **(D)** Visualisation of cell metaclusters for controls (left) and people with CIS/MS (right).

Whole blood phenotyping by flow cytometry with dimensionality reduction identified six presumptive subsets of neutrophils within CD45^+^ cells in blood (**Figure 1A**; metaclusters 13, 14, 15, 16, 18, and 19). All six subsets were CD15^+^CD16^+^, a canonical neutrophil marker combination, and most of the neutrophils formed a single large metacluster (metacluster 13). However, through analysing the expression of the additional cellular markers (**Figure 1B**), five minor neutrophil populations distinct from typical mature neutrophils were observed, categorised as; CD66b^low^ neutrophils (metacluster 19); CD11b^-^ neutrophils (metacluster 16); CD10^low^ neutrophils (metacluster 15); CD62L^low^ neutrophils (metacluster 18); and OLFM4^high^neutrophils (cluster 14). Several CD15^-^ cell metaclusters were present, presumptively attributed to non-neutrophil leukocytes, but not further annotated due to the lack of appropriate lineage-specific markers.

**Figure 2:**
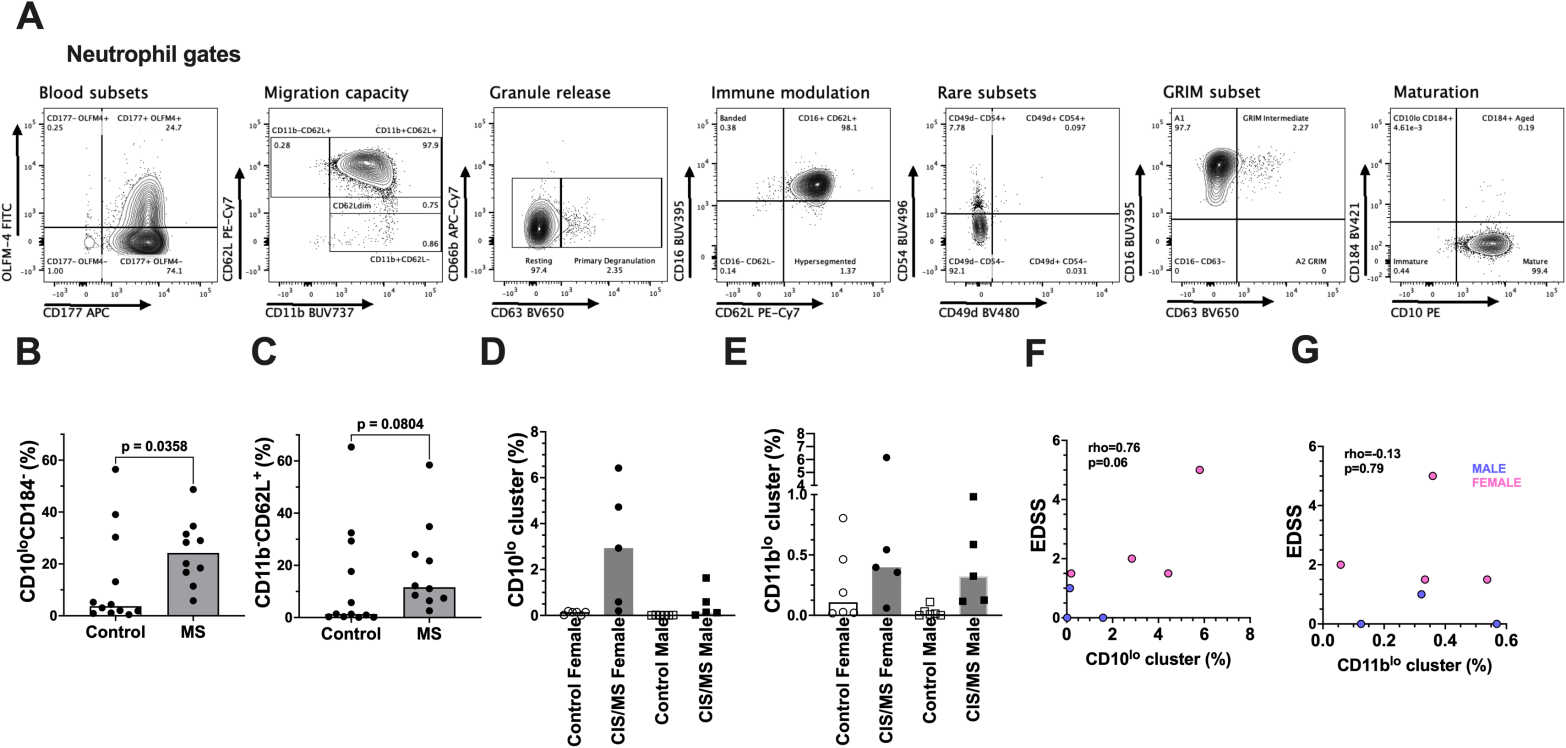
**(A)** Flow cytometry gating strategies applied to CD15^+^CD16^+^ neutrophils identified using the gating strategy shown in **Supplementary Figure 1**. **(B)** Proportions of CD10^lo^CD184^-^ neutrophils (% of total neutrophils) in people with CIS/MS (n=10) and controls (n=12), with median values indicated by the shaded bar and individual values shown as circles; p-value was obtained from a Mann-Whitney test. **(C)** Proportions of CD11b^-^CD62L^+^neutrophils (% of total neutrophils) in people with CIS/MS (n=10) and controls (n=12), with median values indicated by the shaded bar and individual values shown as circles; p-value was obtained from a Mann-Whitney test. Comparison of **(D)** CD10^lo^ and **(E)** CD11b^lo^ neutrophil cluster proportions (% of total neutrophils) in female (circles) and male (squares) participants in control (open shapes) and CIS/MS (closed shapes) groups. Data were available from n=6 control females, n=5 CIS/MS females, n=6 control males, and n=5 CIS/MS males. Correlation between proportions of **(F)** CD10^lo^ and **(G)** CD11b^lo^ neutrophils clusters in blood (as a proportion of total neutrophils) and clinical EDSS scores at the time of sampling. Spearman rho values and p-values are shown for n=7 individuals with CIS/MS with an EDSS score within 1 month prior to the time of blood collection; data points from males are coloured blue, data points from females are coloured pink.

Notably, the CD10^low^ (cluster 15) and CD11b^low^ (cluster 16) neutrophil subsets were significantly enriched in people with CIS/MS compared to controls, both as a proportion of CD45^+^ cells and as a proportion of neutrophils (**Figures 1C, D**; **Supplementary Figure 2**). In clustering analyses, we identified that CD11b^-^ cells that were not captured in our original gating strategy; therefore, these cells were gated and included in subsequent manual gating of neutrophils (**Figure 2A**). Using conventional gating (**Figure 2A**), we confirmed that the proportion of immature CD10^low^CD184^-^ neutrophils, but not CD11b^-^CD62L^+^ neutrophils, was significantly increased in the blood of people with CIS/MS compared with controls (**Figures 2B, C**). There was substantial overlap between the CD10^low^ and CD11b^-^ populations when Boolean gating was applied, though not all CD10^low^CD184^-^ neutrophils were CD11b^-^CD62L^+^ (median 25% across all study participants), and likewise, not all CD11b^-^CD62L^+^ neutrophils were CD10^lo^CD184^-^ (median 43%). The proportion of immature CD10^low^CD184^-^ neutrophils within the CD11b^-^CD62L^+^ gate was significantly higher (p=0.003), and the proportion of CD11b^-^CD62L^+^ cells within the CD10^low^CD184^-^ neutrophil gate trended to be higher (p=0.06), in people with CIS/MS compared with controls (**Supplementary Figures 3A, B**).

To confirm that CD10 expression was the principal marker that distinguished neutrophils from people with CIS/MS from controls, the relative expression levels of positively expressed blood neutrophils markers (MFI) in people with CIS/MS were compared with controls. There were no significant differences between groups in our set of positive neutrophil markers, though a trend towards lower expression of CD10 on neutrophils from people with CIS/MS was observed (**Supplementary Figure 3C**; p=0.08). To better understand the phenotype of the CD10^low^ neutrophil population, we pooled the control and CIS/MS samples and then performed intra-individual paired comparisons of the surface marker expression between the CD10^low^CD184^-^ and CD10^+^CD184^-^ (main) neutrophil populations. Excluding markers that were not strongly expressed in blood neutrophils, the median expression of several neutrophil markers was significantly lower on CD10^low^CD184^-^ neutrophils (**Supplementary Figure 3D**). In particular, the MFI of CD16 and CD11b (and CD10, as expected) was approximately halved on CD10^low^CD184^-^ neutrophils compared with their CD10^+^CD184^-^ counterparts. The differences between groups of other markers such as CD177, while statistically significant, were much smaller and may be of limited biological relevance. This suggests that these CD10^lo^ neutrophils align with a previously described immature phenotype (16), and substantially overlap with, but are not identical to, the CD11b^-^ population.

### 3.2 Associations between CD10^low^ neutrophil frequencies and clinical characteristics

Since we identified that CD10^low^ and CD11b^low^ neutrophil clusters were associated with CIS/MS, we examined whether any explanatory clinical variables contributed to inter-individual variation in these populations. Exploratory analyses did not identify any significant differences in CD10^low^ or CD11b^low^ neutrophil cluster prevalence between females with CIS/MS and males with CIS/MS, or between control females and control males. However, the highest median values in this small cohort were observed in females with CIS/MS and the lowest in control males (**Figures 2D, E**).

Using Spearman correlation, a trend towards a positive association was observed between the proportions of CD10^low^ neutrophils and EDSS scores in participants with CIS/MS within 1 month of blood collection (n=7; p=0.06; **Figure 2F**). There did not appear to be any relationship between CD11b^low^ neutrophil proportions and EDSS scores (**Figure 2G**). Females with CIS/MS appeared to have higher EDSS scores, which suggested that the relationships between immature neutrophils, sex, and EDSS scores could be confounded by sex. A linear regression model was used to explore the potential impacts of age, sex, and EDSS on CD10^low^ neutrophil cluster frequencies in blood in people with CIS/MS. After adjusting for the potential interaction between sex and EDSS, the association between EDSS and CD10^low^ neutrophil frequency remained suggestive of a positive association but did not reach statistical significance (**Table 2**; p=0.08).

**Table 2:** Outcomes of linear regression model for blood CD10^low^ neutrophil cluster frequencies as the outcome variable in n=7 people with CIS/MS with available EDSS assessment data.

Explanatory variable	Estimate	Standard error	P-value
Age	-0.010	0.061	0.87
Male Sex	0.30	1.54	0.85
EDSS	1.19	0.55	0.082
Male Sex *EDSS	-1.27	0.74	0.15

Explanatory terms in the model were EDSS, age, sex, and a sex*EDSS interaction term. Estimates reported in the table show unstandardised regression coefficients (β).

### 3.3 Other markers of blood neutrophil subsets

Finally, we investigated circulating neutrophils through conventional gating for previously published neutrophil subset composition (13), with the initial gating of neutrophils slightly modified due to different allocation of fluorophore to CD66b. Populations of interest included: olfactomedin 4 (OLFM4)-positive neutrophils (17), thought to more readily undergo NETosis (18); CD63-expressing neutrophils, suggestive of primary granule release (19); tissue-experienced neutrophils that have returned to the bloodstream, a rare subset identified by CD54^+^ expression (20); CD49d^+^ neutrophils, a rare subset reported to develop in people with atopy (21, 22); banded neutrophils, another proposed approach to identify immature neutrophils through CD16 and CD62L expression (23, 24); aged neutrophils identified by upregulation of CXCR4 expression (25–27); and granule releasing immunomodulatory ‘GRIM’ neutrophils (28). These are populations that have been covered in multiple reviews of neutrophil subsets (12, 29, 30) and observed to be altered across various diseases and tissues. Understanding of these subsets is now being informed by new single cell RNAseq-based analyses, which are delineating further subtleties in neutrophil composition (31). Beyond CD10^low^ neutrophils, we did not find any differences in these other populations between controls and people with CIS/MS (not shown).

## 4 Discussion

Recent studies suggest that neutrophils may be involved in autoimmune diseases, including MS (5, 6, 8–11). Yet it is not clear whether a discrete neutrophil population is of particular importance for disease activity in MS. This study sought to investigate the abundance and phenotypes of neutrophil subpopulations reported in scientific literature in a cohort of people with recent CIS/MS onset. Using high-dimensional clustering analysis, we found that people with recent CIS/MS demyelinating events had increased proportions of circulating immature neutrophils (CD10^low^ and CD11b^low^ clusters) compared with controls. Furthermore, we identified a trend toward positive correlation between CD10^low^ neutrophil proportions and MS severity, suggesting they could contribute to MS, or be a biomarker of disease activity. In line with recently proposed naming conventions for neutrophils (31), enriched neutrophils in this study were designated ‘immature CD10^low^ blood neutrophils’. While we did not investigate their functional properties, similar CD10^low^ neutrophils were reported by others to promote T cell survival, proliferation, and IFN-γ production by T cells (16). T cells are a key modulator of immune responses, and the dysregulation of T cell function is an important mediator of MS neuropathology. However, the functional roles of immature CD10^low^ blood neutrophils in context of MS remains to be examined.

The presence of increased proportions of immature neutrophils in blood warrants further investigations into factors that drive their migration into the blood in MS. In people with MS, increased bone marrow neutrophil counts and myeloid-cell progenitors has been reported (32), which may relate to increased immature neutrophil levels in blood. Previous research has shown that CD10^low^ neutrophils are released into the blood of people treated with G-CSF (16) and are systemically increased following *in vivo* inflammatory challenges (33). GM-CSF may be an important chemokine for recruiting neutrophils to the brain, as CSF of people with MS contains significantly higher levels of GM-CSF than in controls (34). In addition, GM-CSF-expressing T cells and B cells are enriched in the blood of people with MS compared with controls (35–37). Therefore, it is possible that neutrophils with immature phenotypes are attracted by cytokine-secreting T and B cells involved in MS disease pathogenesis, as recently reported in experimental autoimmune encephalomyelitis (EAE) (38). The relationships between chemokines and immune cells in context of disease activity in MS should be investigated by future research.

Both clustering and traditional gating of neutrophils identified a significantly increased proportion of CD10^low^ neutrophils in people with CIS/MS, supporting their potential importance in MS pathology. While clustering identified CD11b^-^ neutrophils as a separate population increased in proportion in people with CIS/MS, we found with conventional gating that the CD10^low^ and CD11b^low^ populations were substantially correlated in many individuals. This may partially explain why dimensionality reduction identified a numerically smaller population of CD10^low^ neutrophils in these samples than conventional gating of immature neutrophils. This suggests that CD10^low^ neutrophils are not uniform in expression of other markers, and markers with greater expression variation had a stronger influence on clustering in this dataset. A recent report of increased CD10 protein expression in total neutrophils isolated from people with established MS compared with controls contrasts with this finding (39); however, differences in modalities mean we assessed only surface CD10, compared to proteomic measurement of both intracellular and surface CD10. This could underlie the discrepancy, along with differences in cohort characteristics such as participant age and disease stage, with our cohort more reflective of the initial stage of disease.

We examined neutrophils identifiable using the gating methods described for this flow cytometry panel (13), canvassing several reported neutrophil populations (e.g. ‘aged’ or ‘primed’ neutrophils) (25–27). Low-density neutrophils (LDN), described to be enriched in peripheral blood mononuclear cell fractions from people with MS (40), were not assessed in this study of whole blood, however, LDN could share features with immature neutrophil populations. Increased frequencies of neutrophils are reported in blood of MS and other demyelinating diseases including NMOSD (41); whether CD10^low^ neutrophils are comparably enriched in other demyelinating disease could be addressed by future studies. Although we cannot exclude the possibility that the small size of our cohort prevented further findings, and that recent corticosteroid exposure in two participants could have impacted neutrophil phenotypes in those individuals, we suggest that future research efforts into neutrophils during MS should focus on the functions of immature CD10^low^ neutrophils in larger validation cohorts. There has been recent discussion on neutrophil identification, including the definition of “immature” neutrophils (31). While our study was conducted before publication of this new roadmap, future MS research efforts to characterise neutrophils should seek to consolidate approaches to neutrophil definitions as well. CNS neutrophils may include, or be enriched for, additional neutrophil phenotypes not observed in this work. There are limitations of using a k-means clustering approach for cell subset analysis, including errors potentially introduced by scaling and outliers (14, 42), and in our study we were unable to correct for batch effects due to the sporadic nature of our cohort patient presentation. To account for this, data were only included in analyses if they met strict criteria for target channel PMT values on each run, to minimise potential variability introduced by batch across samples. We also strictly included only samples that could undergo analysis within three hours of collection, which was a challenge for our opportunistic recruitment at an off-site clinic. While these stringent inclusion quality control criteria necessarily limited the cohort size, they prioritised biological reliability of neutrophil populations identified. The equal number of males and females in the MS cohort of this study differs from the known female predominance of MS, and likely reflects the modest cohort size; larger studies will be needed to examine whether neutrophil phenotypes in people with MS differ by sex. In addition, we have not studied people with MS with no evidence of disease activity or on disease-modifying therapies; studying CD10^low^ immature blood neutrophils in this context could clarify their relationship to disease activity and may assist in understanding relapse events.

In summary, using an optimised neutrophil flow cytometry panel to identify key previously described neutrophil populations, this study demonstrated that CIS/MS is associated with an increased proportion of immature CD10^low^ blood neutrophils, suggesting a possible role in contributing to pathogenesis, or potential as a biomarker of MS disease activity. While neutrophils may not contribute directly to antigen-specific autoimmune responses, they are the most abundant immune cell in blood and are capable of amplifying existing immune responses and impacting T and B cells involved in MS pathogenesis. The factors driving the migration, expansion, and the functional properties of these cells, as well as their relationship with disease activity in context of MS are yet to be determined and represent important areas for future investigation for the field.

## Data Availability

The raw data supporting the conclusions of this article will be made available by the authors, without undue reservation.
