# Supplementary material for: Immature CD10low blood neutrophils are enriched in people with multiple sclerosis
Source: Front Immunol. 2026 Apr 29;17:1782621. doi: 10.3389/fimmu.2026.1782621 (PMC13167547; doi:10.3389/fimmu.2026.1782621)
Supplement: Supplementary file 1 [file DataSheet1.pdf]

Supplementary Material

1 Supplementary Figures and Tables

1.1 Supplementary Tables

Supplementary Table 1. Details of the flow cytometry antibody panel. *Antigens whose fluorochromes differ from Schofield et al. 2024.

**Surface Panel**						
**Antigen**	**Clone**	**Fluorochrome**	**Product code**	**Supplier**	**Species**	**Isotype**
CD16	3G8	BUV395	563785	BD Biosciences	Mouse	IgG1, κ
CD54 (Optibuild)	HA58	BUV496	741152	BD Biosciences	Mouse	IgG1, κ
CD11b (Optibuild)	ICRF44	BUV737	748588	BD Biosciences	Mouse	IgG1, κ
CD184	12G5	BV421	566282	BD Biosciences	Mouse	IgG2a, κ
CD49d	9F10	BV480	566183	BD Biosciences	Mouse	IgG1, κ
L/D FVS575v*	N/A	BV605	565594	BD Biosciences	N/A	N/A
CD63	H5C6	BV650	353026	BioLegend	Mouse	IgG1, κ
CD15	W6D3	BV786	323043	BioLegend	Mouse	IgG1, κ
CD10	HI10a	PE	561002	BD Biosciences	Mouse	IgG1, κ
CD62L	DREG-56	PE/Cy7	304821	BioLegend	Mouse	IgG1, κ
CD177	MEM-166	APC	315807	BioLegend	Mouse	IgG1, κ
CD45*	HI30	AF700	566962	BD Biosciences	Mouse	IgG1, κ
CD66b*	QA17A51	APC/Fire 750	396907	BioLegend	Mouse	IgG1, κ
**Intracellular Panel**						
**Antigen**	**Clone**	**Fluorochrome**	**Product code**	**Company**	**Species**	**Isotype**
OLFM4	12	AF488	NBP3-06415- & 3320005 (Lightning link)	Novus	Mouse	IgG1

1.2 Supplementary Figures

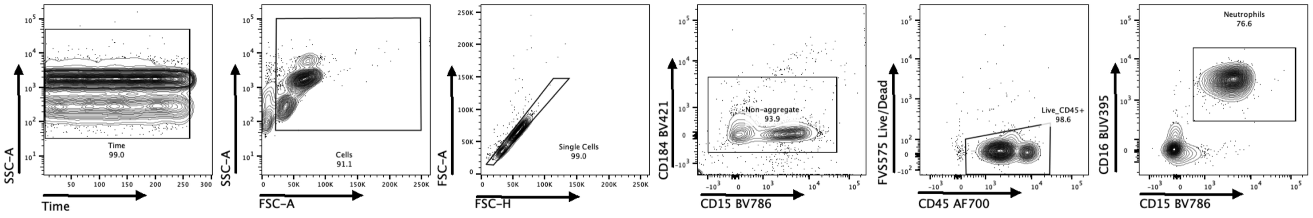

Supplementary Figure 1. Representative plot of flow cytometry gating approach to identify neutrophils (CD15^{+}/CD16^{+}). Cells were gated in a Boolean approach using gates from left to right on time, size, singlet cells, non-aggregate, Live CD45+ cells, and finally on neutrophils.

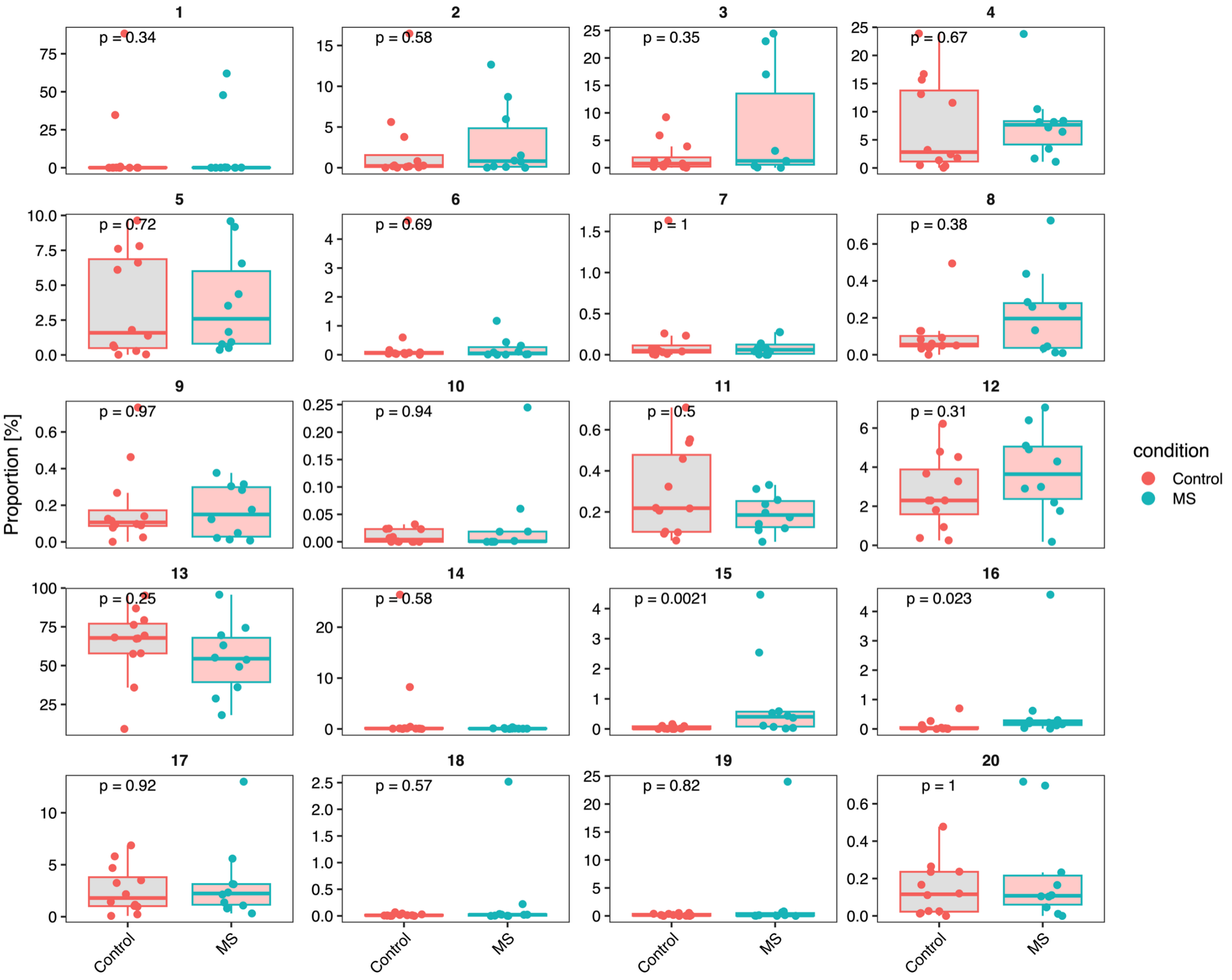

Supplementary Figure 2. Relative proportions of total CD45$^+$ cell metaclusters identified in controls (n=12) and people with CIS/MS (n=10). Statistical comparisons were made between groups using a two-sided Mann-Whitney test, with resulting p-values shown on the figure.

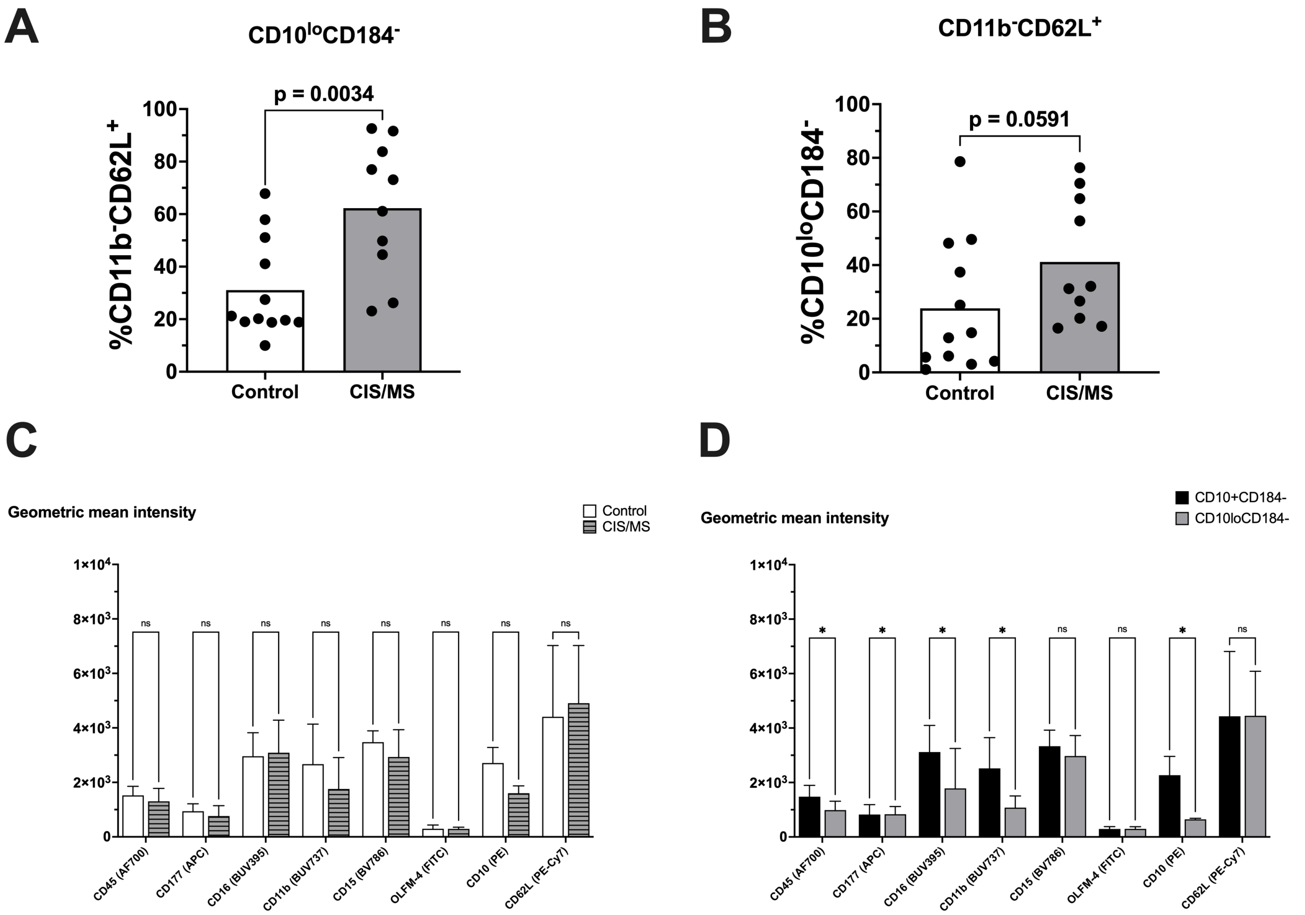

Supplementary Figure 3. (A) Percentage of neutrophils gated as CD11b$^-$CD62L$^+$ that were also CD10^{lo}CD184$^-$ in controls and people with CIS/MS; p-value shown on the figure was derived from a two-sided Mann Whitney test; median values are indicated by the shaded bar and individual values shown as circles. **(B)** Percentage of neutrophils gated as CD10^{lo}CD184$^-$ that were also CD11b$^-$CD62L$^+$ in controls and people with CIS/MS; p-value shown on the figure was derived from a two-sided Mann Whitney test; median values are indicated by the shaded bar and individual values shown as circles. **(C)** Mean fluorescence intensity (MFI) of neutrophil marker expression on total neutrophils in people with CIS/MS (n=10) or controls (n=12), as determined in Figure 1 (CD15$^+$/CD16$^+$). Bar plots show median and interquartile range of group. Comparisons between groups for each marker were made using a two-sided Mann Whitney test. **(D)** Mean fluorescence intensity (MFI) of neutrophil marker expression on CD10$^+$CD184$^-$ vs CD10^{lo}CD184$^-$ neutrophils. Bar

plots show median and interquartile range of group. Comparisons between groups for each marker were made using a Wilcoxon signed-rank test, with Holm-Sidak corrections for multiple comparisons. Resulting p-values plotted above bars showing comparisons. *P-values <0.05; ns=not significant.
